# Association between inactivated influenza vaccine and primary care consultations for autoimmune rheumatic disease flares: a self-controlled case series study using data from the Clinical Practice Research Datalink

**DOI:** 10.1136/annrheumdis-2019-215086

**Published:** 2019-04-29

**Authors:** Georgina Nakafero, Matthew J Grainge, Puja R Myles, Christian D Mallen, Weiya Zhang, Michael Doherty, Jonathan S Nguyen-Van-Tam, Abhishek Abhishek

**Affiliations:** 1 Academic Rheumatology, University of Nottingham, Nottingham, UK; 2 Division of Epidemiology and Public Health, University of Nottingham, Nottingham, UK; 3 Primary Care Centre Versus Arthritis, Keele University, Keele, UK; 4 Nottingham NIHR Biomedical Research Centre, Nottingham, UK

**Keywords:** autoimmune diseases, rheumatoid arthritis, vaccination

## Abstract

To examine the association between inactivated influenza vaccine (IIV) administration and primary care consultation for joint pain, rheumatoid arthritis (RA) flare, corticosteroid prescription, vasculitis and unexplained fever in people with autoimmune rheumatic diseases (AIRDs).

**Methods:**

We undertook within-person comparisons using self-controlled case-series methodology. AIRD cases who received the IIV and had an outcome of interest in the same influenza cycle were ascertained in Clinical Practice Research Datalink. The influenza cycle was partitioned into exposure periods (1–14 days prevaccination and 0–14, 15–30, 31–60 and 61–90 days postvaccination), with the remaining time-period classified as non-exposed. Incidence rate ratios (IRR) and 95% CI for different outcomes were calculated.

**Results:**

Data for 14 928 AIRD cases (69% women, 80% with RA) were included. There was no evidence for association between vaccination and primary care consultation for RA flare, corticosteroid prescription, fever or vasculitis. On the contrary, vaccination associated with reduced primary care consultation for joint pain in the subsequent 90 days (IRR 0.91 (95% CI 0.87 to 0.94)).

**Conclusion:**

This study found no evidence for a significant association between vaccination and primary care consultation for most surrogates of increased disease activity or vaccine adverse-effects in people with AIRDs. It adds to the accumulating evidence to support influenza vaccination in AIRDs.

**Key messages**

**What is already known about this subject?**

- Inactivated influenza vaccine is recommended in patients with autoimmune rheumatic diseases to minimise the increased risk of influenza and its complications in this population.
- Concerns about influenza vaccine associating with increased risk of autoimmune rheumatic disease (AIRD) activity and anecdotal reports of the influenza vaccine triggering diseases such as vasculitis are barriers to seasonal influenza vaccination.

**What does this study add?**

- Seasonal influenza vaccination is not associated with AIRD flare and vasculitis.

**How might this impact on clinical practice or future developments?**

- This study provides new data on the safety of influenza vaccine in people with AIRDs and adds to the accumulating evidence to support seasonal influenza vaccination in this population.

## Introduction

Autoimmune rheumatic diseases (AIRDs) such as rheumatoid arthritis (RA) associate with increased risk of influenza and its complications.^1^ Even though inactivated influenza vaccine (IIV) has clinical and serological effectiveness in AIRDs, its uptake remains suboptimal.^2–4^ For instance, when AIRDs were the sole indication for vaccination, 49% and 59% people aged 18–44 and 45–64 years, respectively, were vaccinated in the 2015–2016 influenza season in the UK,^5^ with lower vaccine uptake reported in Germany.^6^

Barriers to IIV include scepticism about effectiveness, concerns about side effects or disease flare and reports of vaccination triggering AIRDs such as vasculitis.^7–11^ Trials assessing serological response to IIV report stable disease activity following vaccination provided disease-modifying antirheumatic drugs (DMARDs) treatment is continued.^12 13^ However, these studies typically include people with stable disease, and, to the best of our knowledge, a real-world study evaluating the effect of IIV on AIRDs has not been performed. Thus, the objectives of this study were to investigate the association between IIV administration and primary care consultation for joint pain, RA flare, new oral corticosteroid prescription and potential vaccine adverse effects, such as vasculitis and non-infective fever.

## Methods

### Data source

Data were extracted from the Clinical Practice Research Datalink (CPRD).^14^ CPRD is a longitudinal database of anonymised health records of >15 million people registered in >700 general practices in the UK. Participants are representative of the UK population.^14^ It contains details of demographics, diagnoses, immunisations, prescriptions and lifestyle factors.

### Study design

Self-controlled case series (SCCS) was developed for assessing associations between exposures and outcomes using data from participants who develop an outcome of interest and is an accepted methodology for vaccine safety studies.^15 16^ It has the advantage of being unaffected by between-person confounding as each participant acts as their own control. However, it does not account for time varying covariates such as season which vary between the unexposed and exposed periods.

### Source population

Adults aged ≥18 years with RA, spondyloarthritis (SpA) or systemic lupus erythematosus (SLE) and prescribed DMARDs.^3^

### Study period

The study period was 1 September 2006 to 31 of August 2016. This was partitioned into 10 influenza cycles, beginning on 1 September of 1 year and ending on 31 August the subsequent year. Due to the use of non-standard monovalent vaccine alongside trivalent IIV during 2009–2010 pandemic year, data for this year were excluded. Observation periods for each year were censored if death, emigration from general practice or last collection of data from general practice occurred before 31 August of the subsequent year.

### Exposure and outcomes

Vaccination, the exposure of interest, was defined using Read codes^5^ and event dates. Influenza cycles in which a patient was coded as having received the IIV elsewhere, for example, at work for health care professionals or in community pharmacies were excluded from the analysis as the date of vaccination outside primary care is not recorded in the CPRD. For cases with >1 entry for vaccination in an influenza cycle, the earliest record was retained.

The outcomes were primary care consultation for:

- Surrogates of disease activity: joint pain, flare of RA and new corticosteroid prescriptions.
- Vaccine hypersensitivity: vasculitis, non-infective fever. Only the first Read code for vasculitis was considered since it is a chronic illness, and, participants diagnosed with vasculitis before study entry were excluded.

See online supplementary material for details.

10.1136/annrheumdis-2019-215086.supp1 Supplementary data

### Exposure and unexposed periods

**Figure 1:**
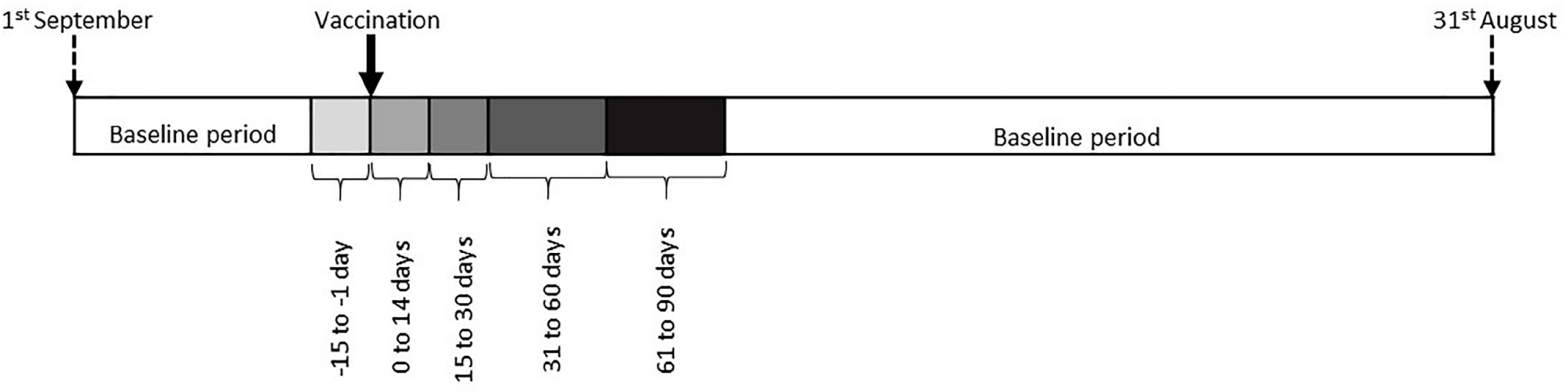
Influenza cycle divided into baseline, prevaccination and postvaccination periods. The baseline extended from latest of diagnosis date or 1st September in index year to 15 days pre-vaccination, and from 90 days post-vaccination to earliest of 31st August of the next year, date of leaving GP surgery, date of death, or latest date of data collection. Exposed period extended from vaccination to 90 days later, and was categorised as illustrated above.

The influenza cycle was divided into unexposed and exposed periods, and the latter was further categorised into smaller time periods (figure 1). The first cut-off was selected at 14-day postvaccination as it takes 2 weeks for the IIV to induce a serological response, and, we hypothesised that this period of immune reconstitution was most likely to associate with disease activity.^17^ The 14-day period immediately preceding vaccination was excluded from the baseline period to minimise confounding due to healthy vaccinee effect.^18^

### Statistical analyses

SCCS involves fitting a Poisson model conditioned on the number of events and calculates incidence rate ratios and 95% CI for each exposure period. Distinct SCCS analysis data sets were drawn for each outcome. Each participant contributed data from one randomly selected eligible influenza cycle in which both vaccination and an outcome of interest occurred (online supplementary table S1). We analysed data from single randomly selected influenza cycle since people who develop an adverse outcome temporally closely related to vaccination, for example, joint pain within 1–2 weeks of vaccination, will be less likely to attend for repeat vaccinations compared to those who have such an event after a longer time period. Thus, if vaccination was to cause an adverse effect, including data from all influenza cycles in which an outcome occurred for a study participant would introduce differential bias. Except for vasculitis, >1 event of the same type in an influenza cycle were considered as recurrent episodes provided the interval between any two consultations was ≥15 days.

As corticosteroids are prescribed for many reasons, we performed sensitivity analysis restricted to corticosteroid prescription on the same day on which there is a primary care consultation for joint pain or RA flare. All analyses were carried out using Stata V.14.

## Results

Data for 14 928 AIRD cases with ≥1 outcome of interest in an influenza cycle in which they received the IIV were included. Of these, 11 953 (80.07%) had RA, 2347 (15.72%) had SpA and 628 (4.21%) had SLE. The majority were female (68.5%) and their mean (SD) age was 59 (14) years. Vaccination associated with fewer primary care consultations for joint pain with the magnitude of reduction broadly consistent across the four risk periods (table 1). When data for the first 30 days were pooled together, vaccination associated with fewer primary care consultations for joint pain (IRR 0.89 (95% CI 0.84 to 0.9)). The 14-day prevaccination period associated with significantly more primary care consultations for joint pain and new corticosteroid prescriptions (table 1). The median (IQR) corticosteroid dose was 10 (5–30) mg/day prednisolone equivalents, data available for 54% prescriptions.

**Table 1:** Association between IIV and consultation for joint pain, new corticosteroid prescription and RA flare

Outcome	Risk period	Events (n)	Person-time (days)	IRR (95% CI)*	P value*
Joint pain	Unexposed	9977	2 712 373	1.00	**<** **0.001**
	15 days prevaccination	788	160 775	**1.29 (1.20 to** **1.39**)	
	Postvaccination intervals				
	0–14 days	479	150 314	**0.84 (0.77 to** **0.92**)	**<** **0.001**
	15–30 days	567	160 842	0.94 (0.86 to 1.02)	0.127
	31–60 days	1121	321 024	**0.93 (0.88 to** **0.99**)	**0.025**
	61–90 days	1069	319 890	**0.90 (0.84 to** **0.96**)	**0.001**
Corticosteroid prescription	Unexposed	9470	2 266 070	1.00	<0.001
	15 days prevaccination	704	135 493	1.21 (1.12 to 1.31)	
	Postvaccination intervals				
	0–14 days	539	126 625	1.00 (0.91 to 1.09)	0.924
	15–30 days	554	135 515	0.96 (0.88 to 1.04)	0.338
	31–60 days	1151	270 109	1.00 (0.95 to 1.07)	0.876
	61–90 days	1182	268 968	1.04 (0.98 to 1.11)	0.184
RA flare	Unexposed	460	153 247	1.00	0.785
	15 days prevaccination	24	8957	0.95 (0.64 to 1.41)	
	Postvaccination intervals				
	0–14 days	27	8400	0.97 (0.65 to 1.45)	0.888
	15–30 days	29	9000	1.12 (0.78 to 1.62	0.525
	31–60 days	59	18 000	1.06 (0.80 to 1.39)	0.693
	61–90 days	46	17 978	0.81 (0.59 to 1.10)	0.180

*Statistically significant results are in bold (p<0.05).

IIV, inactivated influenza vaccine; IRR, incidence rate ratio; RA, rheumatoid arthritis.

There were no significant associations between vaccination and other adverse outcomes in this study (tables 1 and 2). On sensitivity analysis, vaccination was not associated with new corticosteroid prescription on the same day on which there was a primary care consultation for either RA flare or joint pain (online supplementary table S2).

**Table 2:** Association between inactivated influenza vaccine, vasculitis and unexplained fever*

Outcome	Risk period	Events (n)	Person-time (days)	Incidence rate ratio (95% CI)	P values
Vasculitis	Unexposed	89	30 936	1.00	0.906
	15 days prevaccination	5	1815	0.95 (0.38 to 2.33)	
	Postvaccination intervals				
	0–14 days	X^1^	1694	0.41 (0.10 to 1.65)	0.207
	15–30 days	5	1815	0.95 (0.38 to 2.33)	0.906
	31–60 days	8	3630	0.76 (0.37 to 1.56)	0.453
	61–90 days	12	3630	1.14 (0.62 to 2.08)	0.677
Unexplained	Unexposed	92	30 735	1.00	0.507
Fever	15 days prevaccination	X^1^	1819	0.71 (0.26 to 1.94)	
	Postvaccination intervals				
	0–14 days	X^1^	1708	0.71 (0.26 to 1.94)	0.589
	15–30 days	6	1830	1.06 (0.28 to 2.07)	0.887
	31–60 days	5	3650	0.45 (0.18 to 1.10)	0.079
	61–90 days	12	3630	1.09 (0.60 to 1.99)	0.780

X^1^ fewer than five events in each cell, data suppressed according to Clinical Practice Research Datalink policy.

*Unexplained fever was defined as fever not due to a known cause, for example, infection.

## Discussion

This study reports no significant associations between IIV administration and new primary-care corticosteroid prescriptions or primary care consultations for vasculitis and non-infective fever. However, there was a negative association between vaccination and primary care consultations for joint pain upto 90 days postvaccination. Further research is required to understand the underlying mechanism. It is unlikely to result from contextual response or healthy vaccine effect as there is no prevalent belief that vaccination improves AIRD outcomes, and SCCS utilises within-person comparisons accounting for the latter. However, this observation could result from regression to the mean.

We observed increased primary care consultation for joint pain and new corticosteroid prescriptions in the 14 days preceding vaccination. This could indicate opportunistic vaccine promotion to people consulting for AIRD flare. Alternatively, this may be due to the fact that most influenza vaccinations occur in late autumn and winter months^5^ that coincide with increased AIRD activity. It is therefore of interest that the 30-day postvaccination period, also in the late autumn and winter months, expected to have more consultations for joint pain, had significantly fewer consultations.

The potential for vaccines to elicit an immune-mediated adverse reaction has raised concerns about a link between vaccines and AIRDs.^9 10^ However, our data do not identify a significant association between vaccination and vasculitis and are in line with other studies.^19^ Similarly, there was no association between IIV and incident RA in the Epidemiological Investigation of Rheumatoid Arthritis cohort.^20^

The main strength of this study is its robust design, employing the SCCS method. By performing within-person comparisons, it minimises the influence of confounding between individuals, a serious problem in observational studies of adverse-effects following vaccination. Additionally, the use of consultation and prescription data minimised recall bias. The inclusion of a broad spectrum of AIRDs makes our findings generalisable. Additionally, we performed sensitivity analysis restricting to corticosteroid prescriptions on the same day as primary care consultation for either joint pain or RA flare.

However, there are several limitations to this study. First, data on disease activity is not recorded in the CPRD, and primary care consultations occur at least a few days after flare onset. Self-managed flares and those managed by rheumatologists are excluded. Thus, the use of consultation-based database underestimates the event rate. However, these caveats are unlikely to affect the validity of our findings as there is no reason for the ratio between events and primary care consultations to vary across the influenza-cycle. Our a priori decision to restrict the data analysis to one randomly selected influenza-cycle may have reduced the power for some outcomes such as new corticosteroid prescription which occurred in ≥2 influenza cycles for 50% participants. This does not apply to other uncommon outcomes, about 90% of which occurred in only one influenza cycle.

In conclusion, this study supports the safety of influenza vaccine in AIRDs. These data should be used to address the fear of adverse effects from vaccination, an important reason for suboptimal uptake of influenza vaccination in AIRDs.
