# Supplementary material for: Association between inactivated influenza vaccine and primary care consultations for autoimmune rheumatic disease flares: a self-controlled case series study using data from the Clinical Practice Research Datalink
Source: Ann Rheum Dis. 2019 Apr 29;78(8):1122–6. doi: 10.1136/annrheumdis-2019-215086 (PMC6691866; doi:10.1136/annrheumdis-2019-215086)
Supplement: Supplementary data [file annrheumdis-2019-215086supp001.docx]

**Online supplementary material**

**Rationale for outcome selection:** *Surrogates of disease activity:* Initial feasibility assessment of the CPRD revealed that data on AIRD activity scores e.g. DAS-28 are not available in this database. Primary-care consultation for joint pain and primary-care consultations where the GP diagnosed AIRD flare were chosen after reviewing Reports of the OMERACT RA-flare definition working group (J Rheumatol. 2014;41(4):799-809; J Rheumatol. 2011;38(8):1751-8). Fatigue was considered but excluded as there were only 353 consultations for fatigue in a source population of 14,928 AIRD cases suggesting that data about fatigue is not entered by the GPs in their electronic medical records. Fatigue is also not supported as a core outcome measure domain by the OMERACT RA-flare definition working group (Annals of the Rheumatic Diseases 2012;71:1855-1860).

Changes in prescribed treatment also indicate disease flare. Analgesics, NSAIDs, or corticosteroids are used for the initial treatment of flares. As analgesics and NSAIDs are easily obtained over the counter in the UK, and many people with AIRDs keep a ready supply, we chose to use new primary-care corticosteroid prescriptions as a surrogate for disease flare. Changes in Disease Modifying Anti-Rheumatic Drug (DMARD) therapy were not used as a surrogate of disease flare, as, only hospital rheumatologists escalate DMARD therapy, and, any changes in treatment takes 4-6 weeks to reflect in the GP prescriptions.

*Vaccine hypersensitivity*: Given the anecdotal reports of IIV associating with vasculitis, we included vasculitis and non-infective fever as markers of vaccine hypersensitivity. These two outcome measures are also markers of increased systemic AIRD activity which were identified as a core domain for RA-flare by the OMERACT RA-flare definition-working group (J Rheumatol. 2014;41(4):799-809; J Rheumatol. 2011;38(8):1751-8).

**Outcome definition:**

*Joint pain* A Read code list for joint pain was developed. Read codes indicating bone or joint injury, osteoarthritis, haemarthrosis, acute crystal arthritis, septic arthritis, fractures, metastases, tendinopathies were excluded.

*RA flare* A single Read code for RA flare was used to define this outcome.

*New oral corticosteroid prescriptions* were defined as those that are not preceded by a corticosteroid prescription in the preceding 60 days. This is as most primary-care prescriptions in the UK are for 4 weeks, and, a period of 60 days free of corticosteroid prescription excludes people on long-term corticosteroid treatment. Product code lists for corticosteroids were developed, and topical and oral corticosteroids were excluded from this list.

*Non-infective fever* was defined to have occurred if there was a consultation for fever without either

1. antibiotic or antiviral prescription on the same day, or
2. Read codes for acute respiratory, urinary, gastrointestinal tract, skin and soft-tissue, musculoskeletal, nervous system and ear nose and throat infection within the preceding or subsequent 15 days of consultation for fever.

Read code lists for fever, oral antibiotics, oral antivirals and infections were developed to identify these outcomes of interest.

*Vasculitis* Read code lists for vasculitis was developed.

The Read codes used in this study are available from the corresponding author on request.**Supplementary tables:**

**Tables S1** The number of influenza cycles in the study with vaccination and at least one outcome of interest

| **Outcome** | **Number of influenza cycles (%)** | | |
| --- | --- | --- | --- |
|  | **1** | **2** | **> 2** |
| Joint pain | 10,743  (58.60) | 4,406  (24.03) | 3,184  (17.37) |
| Corticosteroid prescription | 9,049  (49.75) | 4,214  (23.17) | 4,925  (27.08) |
| RA flare | 600  (87.46) | 63  (9.18) | 23  (3.35) |
| Unexplained fever | 122  (98.39) | 2(1.61) | 0 |
| Vasculitis | 121  (100) | -/- | -/- |

**Table S2:** The association between Inactivated Influenza Vaccine and new corticosteroid prescription^1^

| **Outcome** | **Risk period** | **No. of events** | **Person-time (days)** | **IRR (95% CI)** |
| --- | --- | --- | --- | --- |
| Corticosteroid prescription^1^ | Unexposed | 258 | 93,448 | 1.00 |
|  | Upto 15 days pre-vaccination | 18 | 5,448 | 1.18 (0.73-1.90) |
|  | Post-vaccination |  |  |  |
|  | 0-14 days | 16 | 5,110 | 1.12 (0.67-1.85) |
|  | 15-30 days | 20 | 5,463 | 1.32 (0.84-2.08) |
|  | 31-60 days | 31 | 10,920 | 1.02 (0.71-1.49) |
|  | 61-90 days | 23 | 10,920 | 0.76 (0.50-1.16) |

^1^ on the same date as a consultation for RA flare or joint pain
